# Combined Analytical and Clinical Performance Evaluation of a Novel Dengue NS1 Rapid Test in a Real-World Endemic Setting

**DOI:** 10.3390/diagnostics16030395

**Published:** 2026-01-26

**Authors:** Jidapa Szekely, Hafik Duereh, Jenureeyah Mongkolprasert, Chadarat Senorit, Wilai Pattoom, Rawadee Suebsaiorn, Sirinda Woraphan, Piyawut Swangphon

**Affiliations:** 1 Faculty of Medical Technology, Prince of Songkla University, Hat Yai 90110, Songkhla, Thailand; 2 Panyananthaphikkhu Chonprathan Medical Center, Pak Kret 11120, Nonthaburi, Thailand; duereh.hafik@gmail.com; 3 K.Bio Sciences Co., Ltd., Klong Luang 12120, Pathumthani, Thailand; jenureeyah@k-biosciences.co.th (J.M.); chadarat@k-biosciences.co.th (C.S.); 4 Somdejphrajaotaksin Maharaj Hospital, Mueang 63000, Tak, Thailand; wilai.taksin@gmail.com (W.P.);

**Keywords:** dengue virus, NS1 antigen, rapid diagnostic test, clinical validation, primary infection, secondary infection

## Abstract

**Objectives**: This study evaluated the analytical and clinical performance of a novel NS1 rapid diagnostic test in a dengue-endemic setting in Thailand. **Methods**: The K-Dengue NS1 Ag test (K.Bio Sciences, Pathumthani, Thailand) was developed. Analytical performance included determination of LOD, reproducibility, and evaluation against potentially cross-reactive pathogens and interfering substances. Unlike conventional assays employing 40 nm colloidal gold, this test incorporates 80 nm gold nanospheres to enhance detection sensitivity. The LOD was determined by serial dilution of recombinant NS1 proteins representing all four dengue virus serotypes. Clinical performance was assessed using 185 archived plasma samples collected between January 2024 and February 2025 from two tertiary care hospitals in Thailand, with a commercial NS1 ELISA serving as the reference standard. **Results**: The K-Dengue NS1 test demonstrated serotype-specific limits of detection (LODs) for recombinant NS1 antigen, 2.9 ng/mL (DENV-1), 0.5 ng/mL (DENV-2), 25.2 ng/mL 27 (DENV-3), and 4.5 ng/mL (DENV-4). Cross-reactivity testing revealed no false positives against closely related arboviruses or common co-infections, and no interference was observed from frequently encountered pathogens or biochemical substances. In clinical evaluation, the assay achieved a sensitivity of 98.08% (51/52), a specificity of 100% (133/133), and an overall accuracy of 99.37%. Importantly, sensitivity was significantly higher in primary infections (100.00%) than in secondary infections (93.3%, *p* = 0.288). **Conclusions**: In this clinically oriented evaluation, the K-Dengue NS1 rapid test showed high specificity and good sensitivity, particularly in primary dengue infections. While the assay may be useful as part of early diagnostic workflows in comparable healthcare settings, reduced sensitivity in secondary infections indicates that negative NS1 results should be interpreted with caution and, where appropriate, supplemented with additional diagnostic methods.

## 1. Introduction

Dengue is a rapidly expanding mosquito-borne viral infection and a major global health threat, particularly in tropical and subtropical regions. An estimated 390 million infections, including 96 million symptomatic cases, occur annually, placing a substantial burden on health systems worldwide [1,2]. Transmitted primarily by *Aedes* mosquitoes, dengue virus (DENV) continues to expand geographically, driven by climate change, urbanization, and global mobility [3,4]. Early, accurate diagnosis is therefore essential for patient management, prevention of severe disease, and timely public health response, especially in resource-limited settings [5].

During the acute febrile phase, usually within the first five days of illness, detection of the dengue non-structural protein 1 (NS1) antigen has proven to be a reliable diagnostic marker. NS1 is a highly conserved glycoprotein that is secreted into the bloodstream at high concentrations early in infection, frequently preceding the appearance of detectable IgM and IgG antibodies [6,7]. Consequently, NS1 detection plays a central role in enabling early diagnosis, thereby facilitating timely clinical decision-making and outbreak response. Rapid NS1-based rapid diagnostic tests (RDTs) employing lateral flow immunoassay (LFA) technology for NS1 detection have been widely adopted because of their simplicity, quick turnaround time, and applicability in decentralized settings [8].

Commercial NS1-based LFAs show variable performance, influenced by serotype, infection status, and assay design [9,10,11]. Key limitations include reduced sensitivity in secondary infections and potential cross-reactivity with flaviviruses such as Zika virus (ZIKV) and Japanese encephalitis virus (JEV) [11,12]. Although ELISA offers higher accuracy, it requires specialized facilities and longer turnaround times, limiting its point-of-care use [13]. Accordingly, there is an urgent need to develop and validate novel NS1 detection platforms that combine high analytical accuracy with the rapidity and ease of LFAs.

Here, we present the analytical and clinical evaluation of a newly developed NS1 rapid diagnostic test (RDT) manufactured by K.Bio Sciences. Analytical performance was assessed through determination of the limit of detection (LOD), reproducibility testing, and cross-reactivity evaluation against related pathogens. Clinical performance was further evaluated using patient samples from dengue-endemic regions of Thailand, with comparative analysis against a reference NS1 ELISA test and a commercial NS1 RDT kit commonly used in clinical laboratories. Our findings provide preliminary insights into the diagnostic performance of this novel assay and suggest potential utility in routine dengue diagnostic settings.

## 2. Materials and Methods

### 2.1. Ethics Statement

The cross-sectional clinical study was conducted according to the Declaration of Helsinki, The Belmont Report, CIOMS Guideline and International Conference of Harmonization in Good Clinical Practice (ICH-GCP) at Srinakharinwirot University from January 2024 to February 2025. Ethical approval was granted by the Human Research Ethics Committee of Panyananthaphikkhu Chonprathan Medical Center, Srinakharinwirot University, Bangkok, Thailand (approval No. EC 029/66, approval date 24 January 2024) and Somdejphrajaotaksin Maharaj Hospital (approval No. EC 13/2566, approval date 3 August 2023).

### 2.2. Development of the NS1 Immunochromatographic Test

#### 2.2.1. Evaluation of Gold Nanoparticle

Three types of gold nanoparticles were evaluated for specificity and sensitivity: 40-nm colloidal gold nanoparticles (K.Bio Sciences, Pathumthani, Thailand), 80 nm gold nanospheres and 150 nm gold nanoshells (Nanocomposix, San Diego, CA, USA). The 40 nm colloidal gold nanoparticles (1 OD) were adjusted to the optimal pH for conjugation. A monoclonal antibody (MAb No. 1) targeting dengue NS1 glycoproteins of all four serotypes (6 µg; Fapon Biotech, Dongguan, China) was conjugated with 1 mL of the colloidal gold suspension. Antibody–gold conjugates were collected by centrifugation, resuspended in borate buffer containing 1% bovine serum albumin (BSA), and adjusted to an optical density (OD) of 20 at 450 nm. The conjugates were then applied to a glass fiber membrane at 1 µL/mm and dried at 37 °C to prepare the conjugate pad.

For the 80 nm gold nanospheres and 150 nm gold nanoshells, covalent conjugation was performed according to the manufacturer’s protocol (Nanocomposix, San Diego, CA, USA). Monoclonal antibodies against DENV1–4 NS1 antigens (MAb No. 1; 20 µg; Fapon Biotech, Dongguan, China) were conjugated with 1 mL of the gold suspension. The conjugates were recovered by centrifugation, resuspended in conjugate storage buffer to the desired OD, applied to glass fiber membranes at 1 µL/mm, and dried at 37 °C.

#### 2.2.2. Assembly of the NS1 Antigen Test Strip

Monoclonal antibodies No. 2 and No. 3 against DENV1–4 NS1 antigens (Total 2.0 mg/mL; Fapon Biotech, Dongguan, China) and goat anti-mouse IgG (1.0 mg/mL; Lampire Biological Laboratories, Pipersville, PA, USA) were dispensed onto nitrocellulose membranes at the test line and control line, respectively. The absorbent pad consisted of untreated cotton paper (Grade 270; Ahlstrom-Munksjö, Helsinki, Finland), and the sample pad was composed of treated cellulose paper (Grade C083; Millipore, Darmstadt, Germany). All pads were assembled with partial overlap to ensure continuous capillary flow of samples and buffer along the strip. Catalog numbers and lot numbers of DENV NS1 ELISA test kit, NS1 antigens and monoclonal antibodies against DENV1–4 NS1 antigens are shown in Supplementary Table S1.

#### 2.2.3. Running Buffer Optimization

To determine the optimal running buffer for the developed lateral flow immuno-assay (LFIA), four buffer systems commonly used in immunodiagnostic applications were evaluated: (i) citrate buffer (50 mM, pH 6.0), (ii) phosphate buffer (50 mM, pH 7.4), (iii) Tris-HCl buffer (50 mM, pH 8.0), and (iv) carbonate-bicarbonate buffer (50 mM, pH 9.5). Each buffer was prepared using analytical-grade reagents (Sigma-Aldrich, St. Louis, MO, USA) and filtered through 0.22 µm membranes prior to use to ensure sterility and particulate-free solutions.

The performance of each buffer was assessed based on the migration rate across the nitrocellulose membrane, signal intensity at the test line, background clarity, and reproducibility of results. For each condition, 10 replicates of positive controls (spiked with recombinant dengue NS1 antigen at 20 ng/mL) and negative controls (buffer only) were tested. Signal intensity was quantified using an ImageJ Gel Analysis tool version 1.54g. The buffer providing the strongest test line intensity with minimal background and consistent flow rate (migration within 15 min) was selected as the optimal running buffer for subsequent assay development.

### 2.3. Limit of Detection (LOD)

To ensure reliable detection of low antigen levels during the early stages of dengue infection, the limit of detection (LOD) of the K-Dengue NS1 Ag Test was determined. Serial dilutions of recombinant dengue virus NS1 proteins representing all four serotypes (Fapon Biotech, Dongguan, China) were prepared, and analytical sensitivity was assessed by comparing test line intensities across antigen concentrations against the baseline at 0 ng/mL. Test line intensities were quantified using the Image Processing and Analysis in Java (ImageJ analysis tool), with the LOD defined as the lowest antigen concentration producing a visible band with quantifiable intensity above background.

### 2.4. Cross-Reactivity and Interference Testing

Analytical specificity was assessed using serum samples spiked with Zika virus (ZIKV) and Japanese encephalitis virus (JEV) NS1 proteins, as well as clinical specimens positive for other arboviruses, including Chikungunya virus (CHIKV). Additional sera from patients with *Plasmodium vivax*, *P. falciparum*, hepatitis B virus, human immunodeficiency virus (HIV), and bacterial infections such as leptospirosis, melioidosis, scrub typhus, and *Salmonella* Typhi were also evaluated. Cross-reactivity was defined as false-positive signals at the test line. All specimens used for cross-reactivity testing were confirmed negative for the dengue NS1 antigen by the Dengue Virus NS1 ELISA Test (Euroimmun, Lübeck, Germany), which served as the reference assay.

Interference testing was performed by spiking NS1-positive and NS1-negative serum samples with commonly encountered pharmaceuticals and biochemical compounds, including acetaminophen, acetylsalicylic acid, amoxicillin, ampicillin, ascorbic acid, caffeine, chloramphenicol, chloroquine, erythromycin, hemoglobin, ibuprofen, nicotinic acid, bilirubin, and triglycerides. Sera with elevated rheumatoid factor and C-reactive protein levels were also tested. All experiments were conducted in accordance with CLSI EP07 guidelines. Each interfering substance was added to dengue-negative serum spiked with 3 × LOD recombinant NS1 antigen (DENV1). Forty microliters of the mixture were applied to the test cassette, and results were recorded after 15 min. All experiments were performed in triplicate.

### 2.5. Clinical Specimens

Between January 2024 and February 2025, a total of 185 archived plasma samples were collected from two tertiary care hospitals in dengue-endemic regions of Thailand: Panyananthaphikkhu Chonprathan Medical Center (Nonthaburi Province) and Taksin Maharaj Hospital (Tak Province). These hospitals serve large populations of patients presenting with acute febrile illnesses.

Leftover specimens were included if they were collected from patients attending healthcare facilities, regardless of the presence of symptoms, and/or presenting with suspected dengue infection within 7 days of fever onset. No restrictions were applied regarding age or gender. Specimens were excluded if leakage, tube defects, or signs of contamination were identified.

EDTA–plasma samples were collected and transported to the laboratory under a maintained cold chain. All specimens were anonymized and relabeled with a new identification code prior to testing. RT-PCR data were not available for this cohort, as samples were collected as part of routine clinical care. The same sample panel was used for NS1, IgM, and IgG ELISA testing as well as for the rapid diagnostic test (RDT). All operators were blinded to all clinical data. ELISA testing and RDT interpretation were performed independently by different operators. Before testing, samples were thoroughly mixed and equilibrated to room temperature. All results were independently interpreted by three trained medical technologists.

Among the collected clinical specimens, 52 were confirmed as NS1-positive and 113 as NS1-negative. Additionally, 20 specimens were obtained from patients with other infections commonly associated with cross-reactivity in dengue antigen-based assays, and 6 were spiked with recombinant arboviral proteins known to exhibit potential cross-reactivity (Table A1). All samples were evaluated using the Dengue Virus NS1 ELISA Test (Euroimmun, Lübeck, Germany) as the reference standard. Comparative performance was assessed between the newly developed NS1 immunochromatographic test (ICT) and the commercial Dengue NS1 Ag test using Bioline™ Dengue Duo (Abbott Diagnostics Korea Inc., Yongin-si, Republic of Korea, Cat. No. 11FK45) as a comparator. The comparator test kit was purchased from a distributor and is routinely used in clinical laboratories. All tests were performed strictly in accordance with the manufacturer’s instructions for use.

Clinical sensitivity, specificity, positive predictive value (PPV), and negative predictive value (NPV) of the K-Dengue NS1 Ag Test were calculated using ELISA results as the reference. Concordance between the K-Dengue test and the commercial NS1 RDT was analyzed, with subgroup analyses stratified by day of illness and classification of primary versus secondary dengue infection (where serological data were available).

### 2.6. Dengue NS1 ELISA

EDTA plasma samples were analyzed for dengue NS1 antigen using the Dengue Virus NS1 ELISA Test (Euroimmun, Lübeck, Germany) following the manufacturer’s instructions. Briefly, patient samples were diluted 1:2 in sample buffer, and 100 μL of the diluted sample was dispensed into each well. After incubation at 37 °C for 60 min, the wells were washed and 100 μL of peroxidase-labeled anti-dengue virus NS1 antibody was added. Following a further 60 min incubation at 37 °C, the plates were washed, and antigen–antibody complexes were visualized by adding 100 μL of substrate solution. After incubation at room temperature for 15 min, the reaction was terminated by adding 100 μL of stop solution, and absorbance was measured at 450 nm using an Infinite 200 pro microplate reader (Tecan, Männedorf, Switzerland).

For semiquantitative analysis, results were expressed as the ratio of the absorbance of the control or patient sample to that of the calibrator. For quantitative analysis, a standard curve was constructed by plotting the absorbance values of the three calibration sera against their assigned unit values using a point-to-point method on a linear–linear scale. The NS1 antigen concentration in patient samples was determined by interpolation from this curve.

### 2.7. Dengue IgM/IgG ELISA

To determine the stage of dengue infection, EDTA plasma samples were tested for IgM and IgG antibodies using the Dengue IgM and IgG ELISA (Euroimmun, Lübeck, Germany) according to the manufacturer’s protocol. Absorbance was measured at 450 nm. Positive determinations were made using the provided IgM and IgG cutoff calibrators.

The IgM/IgG optical density (OD) ratio was calculated by dividing the IgM OD by the IgG OD value. To differentiate between primary and secondary dengue virus infections, an IgM/IgG ratio cutoff of 1.2 was applied, consistent with previously published thresholds [14,15], as summarized in Table A2. Cases with borderline or discordant IgM and IgG ELISA results were conservatively classified as indeterminate and excluded from subgroup analyses comparing primary and secondary infections while remaining included in overall diagnostic performance analyses.

### 2.8. Dengue NS1 Antigen Test

The K-Dengue NS1 Ag Test (K.Bio Sciences, Pathumthani, Thailand) and commercial comparator used are qualitative, membrane-based immunoassays for the detection of dengue NS1 antigen in human serum, plasma, or whole blood. The kit contains a single device for NS1 antigen detection. All assays were performed according to the manufacturer’s instructions. Results were interpreted after 15–20 min. Each cassette contained both a control line and a test line; the appearance of both lines indicated a positive result, whereas the presence of only the control line indicated a negative result.

### 2.9. Data Analysis

Data were tabulated and analyzed using Microsoft Excel (Microsoft Inc., Redmond, WA, USA) and SPSS software (version 22.0; SPSS Inc., Chicago, IL, USA). Descriptive statistics were used to summarize demographic and clinical characteristics. Sensitivity and specificity were reported with 95% confidence intervals (CIs), calculated using the exact binomial (Clopper–Pearson) methods where appropriate.

Comparisons of NS1 positivity between primary and secondary dengue infections were performed using Fisher’s exact test due to small subgroup sizes and the presence of zero cell counts. Effect sizes were quantified using risk ratios, odds ratios, and risk differences, each reported with corresponding 95% confidence intervals, including exact confidence intervals when applicable. Comparisons of NS1 antigen levels between true-positive and false-negative groups were interpreted descriptively.

Sensitivity and specificity of the assays were calculated using a diagnostic test evaluation calculator (MedCalc Software, Version 23.4.8; https://www.medcalc.org/calc/diagnostic_test.php (accessed on 18 August 2024)) based on dengue-positive and dengue-negative samples confirmed by Dengue NS1 ELISA.

## 3. Results

### 3.1. Optimization of Gold Nanoparticle Size and Running Buffer

**Figure 1:**
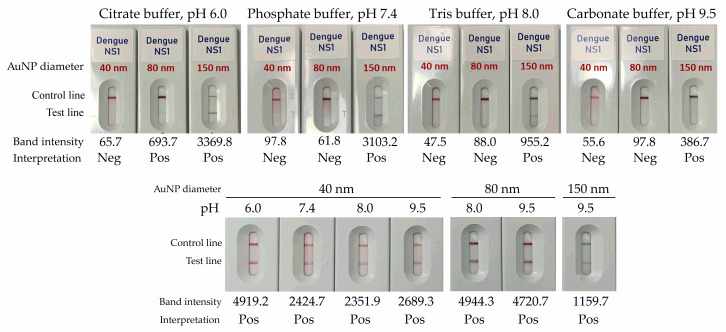
Evaluation of gold nanoparticle size and running buffer conditions. Colloidal gold nanoparticles (40 nm, red), gold nanospheres (80 nm, red) and gold nanoshells (150 nm, dark green) were tested with four running buffers (upper panel) and with NS1-positive serum samples (lower panel). Band intensity at the test line was quantified using the ImageJ analysis tool. Band intensities below 100 were interpreted as negative (Neg).

The performance of gold nanoparticles (AuNPs) with diameters of 40 nm, 80 nm, and 150 nm, each conjugated with anti-dengue NS1 antibodies, was assessed using four running buffers: citrate buffer (pH 6.0), phosphate buffer (pH 7.4), Tris-HCl buffer (pH 8.0), and carbonate–bicarbonate buffer (pH 9.5). As shown in Figure 1, 40-nm AuNPs consistently produced negative results across all buffer systems when tested with buffer alone, indicating adequate specificity under the evaluated conditions. In contrast, 80 nm AuNPs yielded correct negative results when used with Tris-HCl (pH 8.0) and carbonate–bicarbonate (pH 9.5) buffers, while maintaining a clear background and stable migration. Conversely, 150 nm AuNPs generated false-positive signals across all buffer conditions, likely due to nonspecific aggregation or reduced conjugate stability.

When tested with NS1-positive serum samples, all AuNP sizes produced visible positive signals under their respective optimal buffer conditions. However, the 80 nm AuNPs demonstrated the most distinct and consistent test line intensities, underscoring their suitability for assay development (Figure 1). Under these conditions, a carbonate–bicarbonate buffer (pH 9.5) provided optimal signal clarity and stability and was therefore selected for subsequent experiments.

### 3.2. Limit of Detection (LOD) of NS1 of 4 Dengue Serotypes Using the K-Dengue NS1 Ag Test

**Figure 2:**
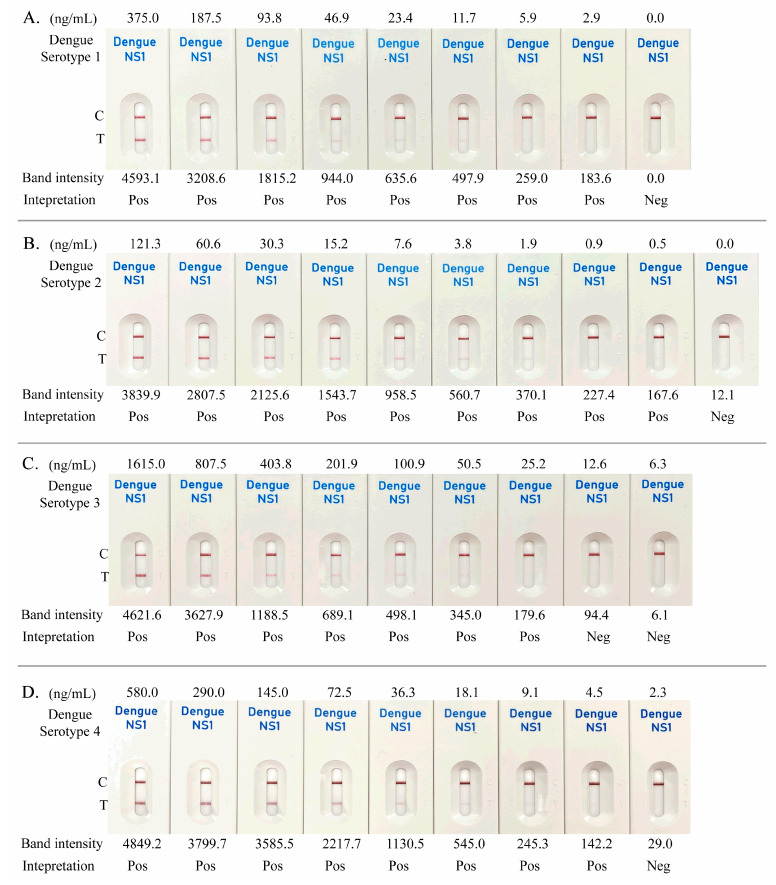
Limit of detection of the K-Dengue NS1 Ag Test using recombinant NS1 proteins of DENV-1 (**A**), DENV-2 (**B**), DENV-3 (**C**), and DENV-4 (**D**). NS1 antigen concentration ranges for each serotype were 375–0 ng/mL (DENV-1), 121.3–0 ng/mL (DENV-2), 1615.0–0 ng/mL (DENV-3), and 580.0–0 ng/mL (DENV-4). Test-line band intensity was quantified using the ImageJ analysis tool; band intensities below 100 were interpreted as negative (Neg).

The analytical sensitivity of the K-Dengue NS1 Ag Test was assessed using recombinant NS1 antigens representing all four dengue virus serotypes. Serial dilutions were prepared across defined concentration ranges for each serotype: 375–0 ng/mL for DENV-1, 121.3–0 ng/mL for DENV-2, 1615.0–0 ng/mL for DENV-3, and 580.0–0 ng/mL for DENV-4. Test line signals were quantified, and band intensities <100 were classified as negative. Based on this threshold, the limit of detection (LOD) was determined to be 2.9 ng/mL for DENV-1, 0.5 ng/mL for DENV-2, 25.2 ng/mL for DENV-3, and 4.5 ng/mL for DENV-4 (Figure 2A–D).

### 3.3. Cross-Reactivity and Interference

To evaluate cross-reactivity, sera spiked with arboviruses (Zika virus [ZIKV] and Japanese encephalitis virus [JEV]) and clinical sera confirmed positive for Chikungunya virus (CHIKV) were tested. Additional sera from patients with other infectious diseases including *Plasmodium vivax*, *P. falciparum*, hepatitis B virus, hepatitis C virus, human immunodeficiency virus (HIV), and various bacterial infections (rickettsiosis, leptospirosis, melioidosis, scrub typhus, and *Salmonella* Typhi)—as well as sera containing rheumatoid factor and elevated C-reactive protein—were also assessed. All 26 samples tested negative, indicating no cross-reactivity or interference.

### 3.4. Clinical Evaluation of the K-Dengue NS1 Ag Test Kit

#### 3.4.1. Dengue NS1 Confirmation by ELISA

All clinical samples included in the evaluation of the antigen test kit were confirmed by Dengue NS1 ELISA. A total of 185 samples were assessed. The average NS1 antigen concentration of positive cases was 193.35 ± 44.91 RU/mL (range: 12.42–231.64 RU/mL). Among dengue-positive cases, 52 samples were obtained from patients with acute symptomatic dengue confirmed by NS1, IgM, and IgG ELISA. The cohort comprised 37.72% males and 62.28% females, with a mean age of 24.8 years (range: 4–72 years). Acute-phase samples were collected within 1–6 days of fever onset. The criteria used to define primary and secondary dengue infections are detailed in Table A2. To differentiate between primary and secondary dengue virus infections, an IgM/IgG ratio cutoff of 1.2 was applied, consistent with previously published thresholds [14,15]. Of the dengue-positive cases, 37 (71.15%) were classified as primary infections and 15 (28.85%) as secondary infections. Dengue-negative cases included 133 samples from patients with non-dengue febrile illnesses, all of which were negative by NS1, IgM, and IgG ELISA.

#### 3.4.2. Clinical Performances of Dengue NS1 Test Kits for Acute Dengue Infection

The sensitivity and specificity of the NS1 antigen-based RDTs (K-Dengue NS1 and the commercial comparator) are summarized in Table 1. Using NS1 ELISA as the reference standard, the sensitivity of K-Dengue NS1 and the comparator was 98.08% and 96.15%, respectively. Both RDT kits demonstrated 100% specificity. The overall accuracy of K-Dengue NS1 and the comparator was 99.37% and 98.73%, respectively (Table 1).

#### 3.4.3. Clinical Performance of the Dengue NS1 Test Kits in Patients with Primary and Secondary Serological Profiles

As NS1 antigen levels vary depending on the infection type, the sensitivity of the NS1-based RDTs was analyzed separately for primary and secondary dengue infections (Table 2). Both RDT kits demonstrated numerically higher sensitivity for primary infections (K-Dengue, 100.00%; comparator, 100.00%) compared with secondary infections (K-Dengue, 93.33%; comparator, 86.67%).

The NS1 positivity rate was 100% in patients with primary infection (37/37; exact 95% CI: 90.5–100%) and 93.3% among those with secondary infection (14/15; exact 95% CI: 68.1–99.9%). Comparisons between primary and secondary infections using Fisher’s exact test yielded *p*-values of 0.288 for the K-Dengue NS1 assay and 0.079 for the comparator assay. Given the limited number of secondary infection cases (*n* = 15) and the resulting wide confidence intervals, Fisher’s exact test reflects low statistical power and imprecision rather than equivalence between groups. Accordingly, these *p*-values are reported descriptively and should not be interpreted as the absence of a difference.

The estimated risk ratio was 1.06 (exact 95% CI: 0.92–1.22) and the odds ratio was 5.29 (exact 95% CI: 0.13–∞). The absolute risk difference was 6.7% (95% CI: −6.0% to 19.3%). Although NS1 positivity tended to be higher in primary infections, the wide and overlapping confidence intervals indicate limited precision, reflecting the small sample size, particularly in the secondary infection group (Table 2).

#### 3.4.4. Clinical Performance of the Dengue NS1 Kits According to the Day After the Onset of Fever

**Figure 3:**
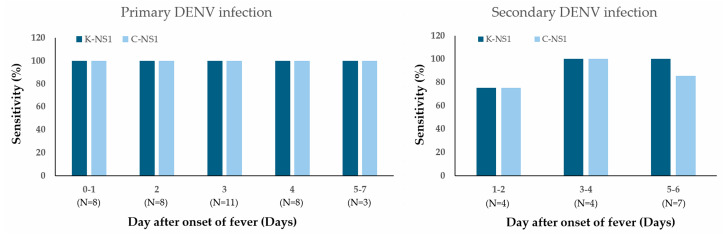
Comparison of the dengue NS1 test kit sensitivity of Dengue NS1 test kits according to the day of fever onset in patients with laboratory-confirmed dengue. Sensitivity is shown for primary infection (**left**) and secondary infection (**right**). Numbers in parentheses below the x-axis indicate the number of dengue patients assessed at each time point.

When stratifying by the day of fever onset, both the K-Dengue NS1 test and the commercial comparator maintained consistently high sensitivity for detecting primary dengue infection throughout the febrile phase. In contrast, the sensitivities of these NS1 tests for secondary infections varied depending on the day after fever onset, indicating temporal differences in antigen detectability between primary and secondary infections (Figure 3).

#### 3.4.5. NS1 Antigen Levels in Dengue-Positive Cases Detected by RDT Kits

Among ELISA-positive samples, quantitative NS1 antigen levels were consistently higher in RDT true-positive cases than in false-negative cases for both evaluated assays (Table 3). For the K-Dengue NS1 assay, the median NS1 antigen level among true-positive samples was 207.2 RU/mL (IQR 192.8–217.0), whereas the single false-negative sample showed a markedly lower value (12.4 RU/mL). Similarly, for the comparator assay, true-positive samples showed a median NS1 level of 207.7 RU/mL (IQR 194.1–217.0), while the two false-negative samples exhibited substantially lower values (12.4 and 25.5 RU/mL; median 18.9 RU/mL). However, given the very small number of false-negative cases, these comparisons are presented descriptively, with emphasis on the magnitude of differences rather than formal statistical inference.

## 4. Discussion

This study provides an in-depth evaluation of the K-Dengue NS1 rapid diagnostic test (RDT), demonstrating its potential as a frontline diagnostic tool for dengue fever in endemic regions. By integrating analytical validation with clinical performance assessment, our findings reinforce the growing body of evidence supporting NS1-based diagnostics while highlighting critical nuances relevant to their deployment in real-world settings.

### 4.1. Analytical Performance and Technical Validation

The K-Dengue NS1 test achieved a limit of detection (LOD) of 2.9 ng/mL for DENV-1, 0.5 ng/mL for DENV-2, 25.2 ng/mL for DENV-3, and 4.5 ng/mL for DENV-4 recombinant NS1 antigens. This analytical evaluation demonstrated serotype-specific differences in the limit of detection (LOD), with a higher LOD for DENV-3 than for DENV-1, DENV-2, and DENV-4. Although these findings confirm detectability across all four serotypes, serotype-specific clinical sensitivity could not be assessed because the clinical specimens were not serotyped. Prior studies from Thailand have reported concurrent circulation of all four serotypes with marked temporal variation, and DENV-3 in particular has shown seasonal and geographic fluctuations in outbreak activity. Distribution of DENV in Bangkok, Thailand, between 2018–2020 showed that DENV-1 has the highest prevalence, followed by DENV-2, DENV-4 and DENV-3 (43.8%, 18.8%, 11.6% and 4.3%, respectively) [16]. In comparison, other parts of Thailand (e.g., the northeastern region) showed a trend of an increased prevalence of DENV-2 over DENV-1 during 2016–2019 [17]. Interestingly, the southern Thai province of Trang exhibited DENV-3 as the predominant serotype in 2024 (*N* = 31, 42.5%), followed by DENV-2 (*N* = 27, 37.0%), DENV-4 (*N* = 13, 17.8%), and DENV-1 (*N* = 2, 2.7%) [18]. In such settings, a higher analytical LOD for DENV-3 may increase the likelihood of false-negative results, especially when NS1 antigen levels are low. Accordingly, the present results should be interpreted as preliminary laboratory evidence of analytical performance. Further clinical validation using RT-PCR–confirmed serotyping is warranted to determine the real-world impact of serotype-specific analytical sensitivity.

This performance is comparable to existing commercial assays and meets the sensitivity requirements for early dengue diagnosis. The use of 80 nm gold nanospheres instead of conventional 40 nm colloidal gold nanoparticles was critical to achieving superior sensitivity, underscoring the importance of nanoparticle selection and conjugation chemistry optimization in lateral flow immunoassays.

Cross-reactivity testing against closely related arboviruses (Zika virus, Japanese encephalitis virus, Chikungunya virus) and other common co-infections demonstrated excellent specificity, with no false-positive results. The absence of interference from commonly encountered pathogens and biochemical compounds supports the robustness of the assay for real-world applications.

### 4.2. Clinical Performance in Real-World Settings

#### 4.2.1. Overall Diagnostic Accuracy

Using NS1 ELISA as the reference standard, the K-Dengue NS1 assay demonstrated high overall sensitivity (98.08%) and specificity (100%). The commercial comparator Bioline™ Dengue Duo (Abbott Diagnostics Korea Inc., Yongin-si, Republic of Korea) showed similar performance, with a sensitivity of 96.2% and specificity of 100%. Together, these results fall within the upper end of the reported performance ranges for NS1 rapid diagnostic tests (RDTs), for which published sensitivities range from 38.6% to 96.2% and specificities from 92% to 100% [19,20,21,22,23].

Reported diagnostic accuracy varies substantially across studies, largely due to differences in study design, patient populations, and reference standards. For example, a Bangkok cohort using nested RT-PCR as the reference reported sensitivity and specificity of 87.88% and 90.00%, respectively, for the SD Bioline™ Dengue Duo assay, highlighting the impact of molecular confirmation criteria on performance estimates [21]. In contrast, surveillance-based studies defining dengue cases using composite reference standards (e.g., RT-PCR, NS1 ELISA, and/or IgM ELISA seroconversion) have reported markedly lower NS1 sensitivities, in some settings as low as 38.6%, underscoring the strong influence of diagnostic reference methods on apparent RDT accuracy [22].

More recently, a large outbreak study from Taiwan using RT-PCR as the reference standard reported a Bioline Dengue NS1 Ag sensitivity of 87.4% and specificity of 100%, with performance varying according to infection status, time from symptom onset, viral load, and serotype distribution [23]. Collectively, these findings indicate that NS1 RDT accuracy is highly context-dependent and influenced by key clinical and virological factors such as infection type, post-symptom onset interval, viral load, and serotype distribution. In that RT-PCR-based evaluation, sensitivity remained substantial in secondary infections (86.8%) and increased markedly in samples collected more than three days after symptom onset (97.4%), supporting the concept that combining NS1 antigen detection with IgM serology or molecular testing can improve overall diagnostic yield. These observations align with our findings that NS1 RDT performance should be interpreted in relation to the clinical context and specimen timing.

#### 4.2.2. Primary Versus Secondary Infection

The K-Dengue NS1 test exhibited numerically higher NS1 positivity in primary (100.0%) compared with secondary infections (93.3%); the limited number of secondary cases resulted in wide confidence intervals and limited statistical power. Similar patterns have been reported in RT-PCR–based evaluations of Bioline Dengue NS1 Ag, where sensitivity in secondary infections remained substantial but varied according to viral load and timing of testing [23]. This aligns with immunopathological mechanisms, where pre-existing antibodies in secondary infections form immune complexes with NS1, thereby reducing detectable antigen levels [24,25,26]. These complexes reduce the concentration of free antigen detectable by capture antibodies, an effect particularly relevant in hyperendemic regions where secondary infections predominate [27]. This immunopathological limitation represents a fundamental challenge to NS1-based assays, necessitating complementary serological or molecular testing for comprehensive case detection.

In dengue-endemic settings where secondary infections with heterologous serotypes are common, serological differentiation between primary and secondary dengue infection is routinely performed using IgM/IgG-based ELISA approaches. Previous studies have demonstrated that IgM/IgG ratio-based classification is a reliable surrogate for infection status across circulating serotypes, independent of the infecting DENV serotype, when appropriately validated cutoffs are applied. In particular, ELISA-based studies have shown that IgM/IgG ratio thresholds provide high sensitivity (≈100%) and specificity (>95%) for identifying secondary infections, reflecting the characteristic anamnestic IgG-dominant immune response following heterologous reinfection [14].

Furthermore, comparative evaluations of dengue duo rapid tests using RT-PCR-confirmed cases across multiple serotypes have consistently applied an IgM/IgG ratio cutoff of 1.2 to distinguish primary from secondary infections, demonstrating stable performance despite variation in serotype distribution and infection timing [15].

In the present study, this cutoff was applied in accordance with published evidence [14]. Cases with borderline or discordant IgM and IgG ELISA results were conservatively classified as indeterminate and excluded from subgroup analyses to minimize misclassification. Nevertheless, in the absence of serotype-confirmed clinical data, the reliability of serological classification should be interpreted cautiously, and future studies integrating RT-PCR–based serotyping are needed to further refine infection status assignment.

#### 4.2.3. Temporal Dynamics of Detection

In primary infections, both assays maintained consistently high sensitivity throughout the acute febrile phase (days 0–7), with only a slight dip observed on day 2. In contrast, sensitivity declined in secondary infections during later days (5–6), reflecting complex host–virus immune interactions. These findings indicate that testing early in the course of illness (within 3–4 days of fever onset) optimizes diagnostic yield, particularly in secondary infections. Our results confirm that NS1 RDTs are most effective during the early febrile phase (days 0–4 post-onset), consistent with the kinetics of NS1 viremia [28]. Sensitivity decreased after day 5 in secondary infections, highlighting the narrow diagnostic window for optimal test use. This aligns with WHO guidelines, which recommend NS1 testing early in the clinical course, supplemented with IgM/IgG or RT-PCR for later phases [29].

### 4.3. Clinical and Public Health Implications

#### 4.3.1. Point-of-Care Utility

The assay’s rapid turnaround (15–20 min), ease of use, and high specificity (100%) make it suitable for point-of-care use in resource-limited settings. However, clinicians should be cautious in interpreting negative results in suspected secondary dengue, where antigen–antibody interactions may lower sensitivity. In such contexts, integration with IgM/IgG assays or RT-PCR can improve diagnostic confidence [26,29].

#### 4.3.2. Surveillance Considerations

The reduced sensitivity in secondary infections could lead to underestimation of disease burden in hyperendemic regions with repeated exposures [27,30]. Surveillance strategies should therefore consider supplementary diagnostic approaches to capture secondary cases more accurately.

### 4.4. Study Limitations

This study has several limitations. First, the overall sample size was modest, with a particularly limited number of secondary infection cases, resulting in wide confidence intervals and limited statistical power for subgroup analyses. Second, the evaluation was restricted to two hospitals within a single country, which constrains the external validity of the findings and limits generalizability to other geographic or epidemiological settings.

Another limitation is the use of NS1 ELISA as the reference standard without RT-PCR and serotype confirmation. In dengue-endemic areas, routine clinical laboratory tests largely support patient care rather than epidemiological surveillance. According to the U.S. Centers for Disease Control and Prevention (CDC), NS1 antigen detection, including NS1 ELISA, is suitable for diagnosing acute dengue infection within the first seven days of illness, while RT-PCR is primarily required for serotype identification and surveillance purposes [31]. Therefore, the use of NS1 ELISA as a reference standard in this research reflects routine clinical laboratory diagnostic procedures. However, using an NS1-based reference standard to evaluate NS1 rapid diagnostic tests carries the risk of data integration bias, potentially leading to overestimation of sensitivity and specificity. To minimize classification errors, borderline ELISA results were excluded from subgroup analyses. Thus, these findings should be interpreted in the context of typical clinical care, and further studies incorporating molecular confirmation are needed, particularly for surveillance-oriented or serotype-specific evaluations. However, ELISA was selected because understanding the dynamics of antibody responses is essential for early diagnosis, clinical management, and epidemiological surveillance, particularly given the increased severity associated with secondary infections [32,33].

Importantly, the practical relevance of this work lies in the need for timely differentiation between primary and secondary dengue infections to reduce morbidity and mortality in endemic regions [34,35].

Finally, pediatric patients and cases of severe dengue were not specifically evaluated. As immune dynamics and viral kinetics may differ in these populations, caution is warranted when extrapolating the results to other clinical groups.

### 4.5. Future Directions

Future research should prioritize multicenter evaluations with larger, demographically diverse cohorts to validate findings across different epidemiological contexts. Comparative studies with RT-PCR would clarify correlations between viral load and NS1 detectability. Technological advances, such as dual-analyte RDTs combining NS1 with IgM/IgG, signal amplification strategies (e.g., fluorescent nanoparticles), or antibody engineering targeting conserved NS1 epitopes, offer promising avenues to enhance sensitivity, especially in secondary infections [28,36]. Cost-effectiveness analyses will also be critical to support large-scale implementation in endemic settings.

## 5. Conclusions

In this single-country, clinically oriented evaluation, the K-Dengue NS1 rapid test demonstrated high specificity and good overall sensitivity, with numerically higher sensitivity observed in primary dengue infections. However, the limited number of secondary infection cases and the absence of molecular confirmation restrict the precision and generalizability of these estimates.

Therefore, the study results should be considered preliminary and interpreted only within the specific clinical context studied. While the assay shows potential for use as part of the initial diagnostic process in similar healthcare settings, broader applicability requires confirmation through large-scale, multi-center studies incorporating diverse patient populations and RT-PCR reference standards. When interpreting NS1-negative results, concurrent testing with molecular assays and/or IgM seroconversion testing remains important to reduce the risk of missed diagnoses.

## Figures and Tables

**Table 1:** Sensitivity and specificity of NS1 rapid diagnostic test kits using NS1 ELISA as the reference standard.

Test Kit	Total (*n*)	% Sensitivity [95% CI]	% Specificity [95% CI]	% Accuracy [95% CI]
K-Dengue NS1	185	98.08% (51/52) [89.74% to 99.95%]	100.00% (133/133) [96.79% to 100.00%]	99.37% [96.62% to 99.98%]
Comparator NS1	185	96.15% (50/52) [86.79% to 99.53%]	100.00% (133/133) [96.79% to 100.00%]	98.73% [95.61% to 99.84%]

**Table 2:** Sensitivity of dengue NS1 rapid diagnostic tests for patients with primary and secondary serological profiles using NS1, IgM, and IgG ELISA as the reference standard.

Infection Type	Total (*n*)	% Sensitivity [Exact 95% CI] ^1^	
		K-Dengue NS1	Comparator NS1
Primary dengue	37	100.00% (37/37) [90.51% to 100.00%]	100.00% (37/37) [90.51% to 100.00%]
Secondary dengue	15	93.33% (14/15) [68.05% to 99.83%]	86.67% (13/15) [59.54% to 98.34%]

^1^ Exact 95% confidence intervals were calculated using the Clopper–Pearson method.

**Table 3:** Sensitivity and specificity of NS1 rapid diagnostic test kits.

Test Kit	NS1 Result	*n*	Quantitative NS1 Antigen Level (RU/mL) ^1^
K-Dengue	True positive	51	Median 207.2 (IQR 192.8–217.0)
	False negative	1	12.4
Comparator	True positive	50	Median 207.7 (IQR 194.1–217.0)
	False negative	2	12.4, 25.5 (Median 18.9, IQR 15.7–22.2)

^1^ Quantitative NS1 antigen level determined by Dengue Virus NS1 ELISA Test (Euroimmun, Lübeck, Germany). Note: Due to the very small number of false-negative samples (*n* = 1–2), NS1 antigen levels are presented descriptively. Confidence intervals and formal statistical comparisons were not considered reliable and are therefore not reported.

## Data Availability

The data may be made available upon reasonable request and subject to approval by the ethics committee. Requests for data access should be directed to the corresponding author and will be reviewed in accordance with ethical guidelines and institutional policies.

## Supplementary Materials

The following supporting information can be downloaded at https://www.mdpi.com/article/10.3390/diagnostics16030395/s1, Table S1: Catalog numbers and lot numbers of DENV NS1 ELISA test kit, NS1 antigens and monoclonal antibodies against DENV1–4 NS1 antigens.

## Abbreviations

The following abbreviations are used in this manuscript:

CHIKV	Chikungunya virus
DENV1	Dengue virus serotype 1
DENV2	Dengue virus serotype 2
DENV3	Dengue virus serotype 3
DENV4	Dengue virus serotype 4
EDTA	Ethylenediaminetetraacetic acid
ELISA	Enzyme-Linked Immunosorbent Assay
HIV	Human immunodeficiency virus
JEV	Japanese encephalitis virus
LOD	Limit of Detection
NS1	Nonstructural protein 1
RDT	Rapid Diagnostic Test
ZIKV	Zika virus

## Appendix A

**Table A1:** Specimens used for analytical and clinical performance evaluations of the K-Dengue NS1 Ag test kit.

Dengue NS1/IgM/IgG-Positive	*N*	Potentially Cross-Reactive Infections	*N*
DENV1 spiked samples	3	*Arboviruses*	
DENV2 spiked samples	3	JEV spiked sample	3
DENV3 spiked samples	3	ZIKV spiked sample	3
DENV4 spiked samples	3	*Other Febrile illnesses*	
*DENV natural infection*		CHIKV natural infection	2
Very early primary infection	29	*Plasmodium vivax*	3
Early primary infection	8	*Plasmodium falciparum*	2
Late primary infection	1	Hepatitis B virus	2
Early secondary infection	15	Hepatitis C virus	1
Late secondary infection	5	HIV	1
Past infection	77	Rickettsia	1
		Leptospirosis	1
		Melioidosis	1
		Scrub typhus	1
		*Salmonella* Typhi	1
		Rheumatoid factor positive sample	2
		C-reactive protein positive sample	2
		Healthy donor samples	30

**Table A2:** Criteria for distinguishing primary and secondary dengue infection.

NS1	IgM	IgG	Fever Onset	Interpretation	*N*
+	−	−	1–3	Very early primary	8
+	+	−	2–5	Early primary	29
−	+	−	>5	Late primary	1
+	+	+	2–4	Early secondary	15
−	+	+	4–6	Late secondary	5
−	−	+	>7 or no symptom	Past infection	77
−	−	−	fever <3 days or no symptom	No infection/ too early to detect	30
