# Supplementary material for: Combined Analytical and Clinical Performance Evaluation of a Novel Dengue NS1 Rapid Test in a Real-World Endemic Setting
Source: Diagnostics (Basel). 2026 Jan 26;16(3):395. doi: 10.3390/diagnostics16030395 (PMC12896629; doi:10.3390/diagnostics16030395)
Supplement: Supplementary file 1 [file diagnostics-16-00395-s001.zip › diagnostics-4081830-supplementary.pdf]

Supplementary documents

Table S1 Catalog numbers and lot numbers of DENV NS1 ELISA test kit, NS1 antigens and monoclonal antibodies against DENV1–4 NS1 antigens

1) DENV ELISA kit details:

Test kits	Company	Catalog number	Lot number
DENV NS1 ELISA	Euroimmun, Lübeck, Germany	EQ 226a-9601-1	E230718AW
DENV IgM ELISA	Euroimmun, Lübeck, Germany	EI 266b-9601 M	E230203RM
DENV IgG ELISA	Euroimmun, Lübeck, Germany	EI 266b-9601 G	E230119BE

2) NS1 antigens details:

NS1 Antigen	Company	Catalog number	Lot number
DENV -1	Fapon Biotech, China	GRCDEN101	20220706
DENV -2	Fapon Biotech, China	GRCDEN102	20211015-1
DENV-3	Fapon Biotech, China	GRCDEN103	20220823
DENV-4	Fapon Biotech, China	GRCDEN104	20220415

3) Monoclonal antibodies against DENV1–4 NS1 antigens:

NS1 Antigen	Company	Catalog number	Lot number
MAb no. 1	Fapon Biotech, China	BRJNS1S107	20210909-1
MAb no. 2	Fapon Biotech, China	BRCNS1S106	20221201-1
MAb no. 3	Fapon Biotech, China	BRCNS1S119	20211224-1
